# Physicians’ views on the role of smoking in smoking-related diseases: findings from cross-sectional studies from 1982–2014 in Estonia

**DOI:** 10.1186/s12971-017-0136-9

**Published:** 2017-07-19

**Authors:** Kersti Pärna, Mariliis Põld, Inge Ringmets

**Affiliations:** Institute of Family Medicine and Public Health, University of Tartu, Ravila 19, 50411 Tartu, Estonia

**Keywords:** Physicians, Knowledge, Attitudes, Smoking, Smoking-related disease, Estonia

## Abstract

**Background:**

Previous reports have shown that physicians who smoke underestimate the effects of smoking on health and this influences their practice. This study was designed to investigate the views of Estonian physicians on the role of smoking in smoking-related diseases.

**Methods:**

Cross-sectional postal surveys were sent to all practising physicians in Estonia in 1982, 2002 and 2014 providing data from 3504, 2694, and 2865 physicians respectively. Data analysis involved calculation of the prevalence of smoking with 95% confidence intervals and calculation of the prevalence of agreement with a causal role of smoking in ischaemic heart disease (IHD), lung cancer and chronic bronchitis. Logistic regression was used to analyse associations between agreement with the statements that smoking is a cause of IHD, lung cancer, chronic bronchitis and study year, smoking status, age group and ethnicity. Fully adjusted odds ratios with 95% confidence intervals were calculated.

**Results:**

The age-standardized prevalence of current smoking among men decreased from 39.6% in 1982 to 14.2% in 2014, and among women from 12.4 to 5.1%, respectively. Compared with 1982, the proportion of physicians agreeing with statements that smoking is a major cause or one of the causes of IHD, lung cancer, or chronic bronchitis was significantly higher in 2002 and 2014. Past and never smokers were more likely to admit a causal role of smoking in lung cancer than smokers. Agreement with a causal role of smoking in IHD and chronic bronchitis was significantly higher among never and past smoking women only. Compared with non-Estonians, the odds ratio indicating agreement with all three statements was significantly higher among Estonians.

**Conclusion:**

From 1982 to 2014, physicians’ attitudes towards the health risks of smoking improved in Estonia. However, their assessment of a causal role of smoking in smoking-related diseases was related to their own smoking habits and ethnicity.

A further decline in smoking among Estonian physicians would require special efforts targeted at physicians. Societal pressure from a national policy could support a further decline in the social acceptability of smoking in Estonia and developments in medical education could provide continuing evidence-based information about the effects of smoking to Estonian physicians.

## Background

Smoking is the leading behavioural risk factor causing a substantial number of preventable deaths worldwide [1]. The main harmful impacts of tobacco use include increased rates of cardiovascular deaths (e.g. caused by ischaemic heart diseases), cancers (e.g. caused by lung cancer), as well as deaths associated with diseases of the respiratory system (e.g. chronic bronchitis) [1, 2]. In Estonia, cardiovascular diseases are the greatest cause of mortality followed by neoplasms [3]. The standardized death rate per 100,000 population from ischaemic heart diseases (I20–I25) was 247.54 and from malignant neoplasm of larynx, trachea, bronchus and lung (C32-C34) was 57.31 in 2014 in Estonia [3].

Physicians play a key role in setting an example by their own smoking in society as well as in encouraging their patients to stop smoking [4–6]. Previous reports conducted on samples of physicians showed that physicians who smoke underestimate the effects of tobacco smoke on health and this influences their practice [4, 7–11]. For effective smoking cessation strategies it is necessary to understand trends in smoking behaviour and attitudes towards smoking among physicians in the country. Also, monitoring trends in smoking behaviour among physicians allows public health policy makers to predict tendencies in smoking among the general population [12]. This study is the first to analyse smoking behaviour and attitudes towards causal role of smoking in smoking-related diseases among Estonian physicians over three decades.

The objective of this study was to investigate the views of Estonian physicians about the role of smoking in smoking-related diseases from 1982 to 2014.

## Methods

### Design and setting of the study

The present study was based on smoking surveys among Estonian physicians carried out in 1982, 2002, and 2014. The methods used in these surveys have been extensively described elsewhere [7, 10, 13–15]. In short, cross-sectional postal surveys using similar, self-administered questionnaires were used to collect information from all practising physicians in 1982 (*n* = 4704), 2002 (*n* = 4140), and 2014 (*n* = 5666) in Estonia. In 1982, all practising physicians were identified from the database of the Ministry of Health in Estonia and in 2002 from the database of Estonian Health Insurance Fund. In these two study years questionnaires were mailed to the physicians’ workplace. In 2014, the sample of all practising physicians was based on data from the Estonian Health Care Professionals Registry and questionnaires were mailed to the physicians’ home addresses. To receive home addresses, data from the Estonian Health Care Professionals Registry were linked with the Population Registry in Estonia.

The number of respondents was 3792 in 1982, 2747 in 2002, and 2903 in 2014. The crude response rates were 80.7, 66.3, and 52.0%, respectively. The corrected response rates (excluding physicians who were unavailable, retired, had an incorrect address, had left Estonia or died) were 67.8% in 2002 and 53.1% in 2014.

### Study variables

**Study years**: 1982, 2002, 2014.

**Smoking status** was determined by combining answers to several questions concerning daily, occasional, past and never smoking status and responses were classified as current (daily or occasional), past and never smoking.

**Attribution of the causal role of smoking in smoking-related diseases** like ischaemic heart disease (IHD), lung cancer, and chronic bronchitis was determined. The answers were classified as agreement if respondents indicated smoking was “a major cause” or “one of the causes”, and disagreement if respondents indicated smoking was “probably not a cause”, “not a cause”, or “cannot say”.

**Age** was measured in full years and analysed in five groups: −34, 35–44, 45–54, 55–64, 65 + .

**Ethnicity** was classified as Estonian/non-Estonian (mainly Russian). Ethnicity referred to self-determined national identity.

### Statistical analysis

As gender was strongly associated with prevalence of smoking, the data was analysed separately for men and women. Data analysis involved the calculation of respondents’ mean age with standard deviation (SD), minimum (min) and maximum (max) value, and calculation of distribution of respondents by age group and ethnicity. The age-standardized prevalence rate of smoking with the corresponding 95% confidence intervals (CI) (using European standard population [16]) and the prevalence of agreement/disagreement with three statements concerning smoking as a possible reason for IHD, lung cancer and chronic bronchitis was calculated.

Associations between agreement (vs disagreement) with a causal role of smoking in IHD, lung cancer, and chronic bronchitis with study year, smoking status, age group, and ethnicity were analysed using logistic regression analysis. Crude and fully adjusted odds ratios (OR) with 95% confidence intervals (CI) were calculated.

Questionnaires where the answers concerning smoking status (six in 1982, twelve in 2002, one in 2014) or attribution of a causal role of smoking in three smoking-related diseases (282 in 1982, 41 in 2002 and 37 in 2014) did not exist, were excluded from the analysis. A total of 3504 questionnaires from 1982, 2694 from 2002, and 2865 from 2014 were used in the analysis. Before the logistic regression analysis, an additional 19 questionnaires (one in 1982, 14 in 2002, four in 2014) that lacked information about ethnicity were excluded from further analysis.

Data were analysed using the statistical package Stata 12 [17].

## Results

The mean age of responding male physicians was 44.4 (SD 12.2, min 23, max 79) and of female physicians 42.4 years (SD 10.1, min 23, max 79) in 1982, 47.8 (SD 12.0, min 25, max 82) and 47.6 (SD 11.2, min 24, max 84) in 2002, and 52.6 (SD 14.5, min 25, max 85) and 51.2 (SD 14.0, min 24, max 86) in 2014, respectively.

Table 1 summarizes the distribution of age groups and ethnicity among Estonian physicians in 1982, 2002, and 2014. The age and ethnicity of physicians varied by study year. While in 1982 the prevalence of young physicians was higher and of old physicians lower, in 2002 and 2014 the prevalence of young physicians was lower and that of old physicians higher. Compared to 1982, the proportion of non-Estonians was lower in 2002 and 2014.

**Table 1:** Distribution of physicians (%) by age groups and ethnicity among Estonian physicians by gender, 1982–2014

Characteristic	1982		2002		2014	
	Male	Female	Male	Female	Male	Female
	*n* = 850	*n* = 2654	*n* = 465	*n* = 2229	*n* = 518	*n* = 2347
Age group						
− 34	26.7	26.3	13.1	13.6	13.5	16.8
35–44	25.9	33.6	32.0	29.2	16.2	14.9
45–54	27.8	27.4	26.9	28.4	23.9	25.6
55–64	14.5	11.2	17.0	22.7	23.8	24.3
65+	5.2	1.6	11.0	6.1	22.6	18.5
Ethnicity						
Estonian	71.4	70.4	80.2	84.5	77.0	83.3
Non-Estonian	28.5	29.7	18.1	15.3	22.8	16.5
Missing answer	0.1	-	1.7	0.3	0.2	0.1

**Fig. 1:**
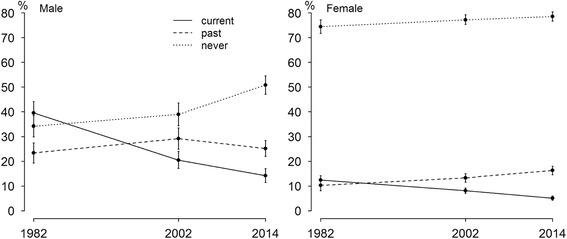
The age-standardized prevalence of current, past and never smoking with 95% CI among Estonian male and female physicians, 1982–2014. Legend: From 1982 to 2014, the age-standardized prevalence of current smoking decreased from 39.6% (95% CI 35.0–44.2) to 14.2% (95% CI 11.4–17.0) among male physicians and from 12.4% (95% CI 10.7–14.2) to 5.1% (95% CI 4.3–6.0) among female physicians (Fig. 1). Age-standardized prevalence of past smoking increased from 23.4% (95% CI 19.3–27.4) to 25.2% (95% CI 22.0–28.4) among male physicians and from 10.3% (95% CI 8.1–12.6) to 16.3% (95% CI 14.6–18.0) among female physicians over the study period. Age-standardized prevalence of never smoking increased from 34.2% (95% CI 29.9–38.5) to 50.8% (95% CI 47.1–54.5) among male physicians and from 74.4% (95% CI 71.7–77.2) in 1982 and 78.5% (95% CI 76.7–80.4) in 2014

The age-standardized prevalence of current smoking decreased from 39.6% (95% CI 35.0–44.2) in 1982 to 20.5% (95% CI 17.2–24.0) in 2002 and to 14.2% (95% CI 11.4–17.0) in 2014 among male physicians and from 12.4% (95% CI 10.7–14.2) in 1982 to 8.1% (7.0–9.2) in 2002 and to 5.1% (95% CI 4.3–6.0) in 2014 among female physicians (Fig. 1). In 1982–2014, the age-standardized prevalence of past smoking increased from 23.4% (95% CI 19.3–27.4) to 25.2% (95% CI 22.0–28.4) among male physicians and from 10.3% (95% CI 8.1–12.6) to 16.3% (95% CI 14.6–18.0) among female physicians over the study period. Age-standardized prevalence of never smoking increased from 34.2% (95% CI 29.9–38.5) to 50.8% (95% CI 47.1–54.5) among male physicians and from 74.4% (95% CI 71.7–77.2) in 1982 and 78.5% (95% CI 76.7–80.4) in 2014.

The majority of physicians agreed with statements that smoking is a major cause, or one of the causes of IHD, lung cancer, and chronic bronchitis (Table 2).

**Table 2:** Attribution of causal role of smoking in smoking-related disease (%) among Estonian physicians by gender, 1982–2014

Statement	1982		2002		2014	
	Male	Female	Male	Female	Male	Female
	*n* = 850	*n* = 2654	*n* = 465	*n* = 2229	*n* = 518	*n* = 2347
Ischaemic heart disease (IHD)						
A major cause	4.94.9	4.8	8.4	7.2	14.7	11.4
One of the causes	79.8	79.3	88.2	89.1	83.8	86.6
Probably not a cause	5.5	4.6	1.9	1.9	0.6	0.9
Not a cause	2.5	1.7	0.4	0.1	0.6	0.1
Cannot say	7.3	9.6	1.1	1.7	0.4	1.0
Lung cancer						
A major cause	12.5	13.5	30.3	32.7	37.6	33.6
One of the causes	77.3	75.1	68.0	66.4	61.2	65.6
Probably not a cause	2.2	2.4	1.3	0.5	0.8	0.4
Not a cause	1.9	1.3	0.2	0.0	0.2	0.1
Cannot say	6.1	7.8	0.2	0.5	0.2	0.3
Chronic bronchitis						
A major cause	24.4	23.0	33.6	35.9	38.6	40.3
One of the causes	70.4	70.9	65.2	63.2	60.4	58.9
Probably not a cause	1.5	0.9	0.9	0.5	0.4	0.3
Not a cause	1.1	0.9	0.2	0.1	0.0	0.2
Cannot say	2.7	4.3	0.2	0.4	0.6	0.3
Total	100	100	100	100	100	100

In the adjusted logistic regression model, agreement with the statement that smoking is a cause of IHD was associated with study year and ethnicity among male and female physicians, and with smoking status and age among female physicians only (Table 3). Compared to the first study year, agreement with this statement was significantly higher among male and female physicians in 2002 and 2014. In comparison with non-Estonians, Estonian physicians agreed significantly more that smoking is a cause of IHD. Among female physicians, attributing a causal role of smoking in IHD was associated with smoking status and age. Compared to currently smoking females, agreement with this statement was significantly higher among past and never smokers. Compared to under 35-year-old female physicians, agreement with the statement was slightly lower in the age groups 45–54, 55–64, and 65+. After adjustment, smoking status and age appeared not to be associated with attribution of a causal role of smoking in IHD among male physicians.

**Table 3:** Agreement (vs disagreement) with the statement that smoking is a cause of the ischaemic heart diseases (IHD) among Estonian male and female physicians

Characteristic	Male physicians		Female physicians	
	Crude OR (95% CI)	Adjusted OR^a^ (95% CI)	Crude OR (95% CI)	Adjusted OR^a^ (95% CI)
Study year				
1982	1	1	1	1
2002	**4.98 (2.93– 8.49)**	**4.49 (2.61– 7.72)**	**5.00 (3.91–6.39)**	**5.03 (3.91– 6.48)**
2014	**11.50 (5.58–23.70)**	**10.40 (4.95–21.85)**	**9.24 (6.80–12.56)**	**9.78 (7.04–13.59)**
Smoking status				
Current	1	1	1	1
Past	**1.84 (1.21–2.79)**	1.16 (0.74–1.82)	**2.64 (1.89–3.69)**	**1.66 (1.17–2.35)**
Never	**2.21 (1.50–3.27)**	1.43 (0.95–2.15)	**1.95 (1.55–2.45)**	**1.63 (1.28–2.08)**
Age				
− 34	1	1	1	1
35–44	1.18 (0.75–1.84)	0.96 (0.60–1.53)	0.99 (0.77–1.28)	0.96 (0.74–1.25)
45–54	**1.72 (1.07–2.78)**	1.26 (0.76–2.08)	0.94 (0.74–1.21)	**0.72 (0.56–0.94)**
55–64	1.63 (0.96–2.76)	1.01 (0.58–1.76)	1.28 (0.96–1.71)	**0.70 (0.52–0.96)**
65+	**2.64 (1.29–5.38)**	1.00 (0.46–2.15)	**1.84 (1.20–2.81)**	**0.56 (0.35–0.89)**
Ethnicity				
Non-Estonian	1	1	1	1
Estonian	**2.20 (1.56–3.10)**	**1.88 (1.31–2.69)**	**1.84 (1.52–2.22)**	**1.35 (1.10–1.64)**

^**a**^Each OR was adjusted for all other characteristics in the table

Data in bold shows significant differences

Agreement with the statement that smoking is a cause of lung cancer was associated with study year and smoking status among male and female physicians and with ethnicity among female physicians only (Table 4). Compared to the first study year, agreement with this statement was significantly higher in 2002 and 2014. Compared to current smokers, agreement with this statement was higher among past and never smokers. Compared to non-Estonian female physicians, Estonian ones had 1.41 times higher probability of agreeing that smoking is a cause of lung cancer. After adjustment, ethnicity appeared not to be associated with attribution of a causal role of smoking in lung cancer among male physicians.

**Table 4:** Agreement (vs disagreement) with the statement that smoking is a cause of lung cancer among Estonian male and female physicians

Characteristic	Male physicians		Female physicians	
	Crude OR (95% CI)	Adjusted OR^a^ (95% CI)	Crude OR (95% CI)	Adjusted OR^a^ (95% CI)
Study year				
1982	1	1	1	1
2002	**6.41 (3.08–13.34)**	**5.72 (2.72–12.05)**	**12.94 (8.36–20.03)**	**12.91 (8.27–20.15)**
2014	**9.72 (4.22–22.41)**	**7.70 (3.25–18.27)**	**15.83 (9.92–25.25)**	**16.54 (10.06–27.17)**
Smoking status				
Current	1	1	1	1
Past	**2.86 (1.69–4.84)**	**1.90 (1.09–3.32)**	**3.87 (2.68–5.60)**	**2.29 (1.55–3.38)**
Never	**3.39 (2.09–5.51)**	**2.31 (1.39–3.82)**	**4.20 (3.28–5.39)**	**3.71 (2.84–4.83)**
Age				
− 34	1	1	1	1
35–44	0.77 (0.45–1.33)	0.58 (0.33–1.02)	**1.43 (1.05–1.93)**	**1.41 (1.03–1.94)**
45–54	1.60 (0.87–2.97)	1.08 (0.57–2.04)	1.22 (0.91–1.63)	0.82 (0.60–1.12)
55–64	1.42 (0.73–2.78)	0.81 (0.40–1.63)	**1.82 (1.28–2.59)**	0.81 (0.56–1.19)
65+	2.38 (0.95–5.94)	0.74 (0.28–1.97)	**3.17 (1.76–5.72)**	0.66 (0.35–1.26)
Ethnicity				
Non-Estonian	1	1	1	1
Estonian	**1.87 (1.23–2.84)**	1.49 (0.96–2.30)	**2.01 (1.60–2.53)**	**1.42 (1.11–1.82)**

^**a**^Each OR was adjusted for all other characteristics in the table

Data in bold shows significant differences

Agreement with the statement that smoking is a cause of chronic bronchitis was associated with study year and ethnicity among male and female physicians and with smoking status among female physicians only (Table 5). Compared to 1982, physicians agreed with this statement significantly more in 2002 and 2014. Estonian physicians agreed significantly more than non-Estonian physicians that smoking is a cause of chronic bronchitis. Compared to currently smoking female physicians, agreement was significantly higher among past and never smoking females. After adjustment, smoking status appeared not to be associated with attribution of a causal role of smoking in chronic bronchitis among male physicians.

**Table 5:** Agreement (compared to disagreement) with the statement that smoking is a cause of chronic bronchitis among Estonian male and female physicians

Characteristic	Male physicians		Female physicians	
	Crude OR (95% CI)	Adjusted OR^a^ (95% CI)	Crude OR (95% CI)	Adjusted OR^a^ (95% CI)
Study year				
1982	1	1	1	1
2002	**4.21 (1.78– 9.94)**	**3.95 (1.65– 9.46)**	**6.50 (4.15–10.19)**	**6.26 (3.95– 9.91)**
2014	**5.73 (2.26–14.53)**	**5.05 (1.91–13.33)**	**8.40 (5.15–13.72)**	**8.23 (4.89–13.84)**
Smoking status				
Current	1	1	1	1
Past	**1.88 (0.98–3.63)**	1.38 (0.69–2.76)	**2.69 (1.63–4.45)**	**1.68 (4.30–10.36)**
Never	**2.61 (1.37–4.95)**	1.88 (0.97–3.65)	**2.45 (1.74–3.46)**	**2.11 (1.48– 3.01)**
Age				
− 34	1	1	1	1
35–44	0.53 (0.24–1.18)	0.43 (0.19–0.96)	1.20 (0.80–1.80)	1.14 (0.75–1.73)
45–54	1.02 (0.43–2.45)	0.72 (0.29–1.76)	1.00 (0.68–1.48)	0.74 (0.50–1.10)
55–64	0.81 (0.33–2.03)	0.51 (0.20–1.32)	1.27 (0.81–1.99)	0.69 (0.43–1.10)
65+	1.35 (0.41–4.45)	0.53 (0.15–1.88)	**2.51 (1.18–5.36)**	0.80 (0.36–1.77)
Ethnicity				
Non-Estonian	1	1	1	1
Estonian	**2.23 (1.29–3.84)**	**1.88 (1.08–3.30)**	**2.34 (1.75–3.13)**	**1.72 (1.27–2.32)**

^**a**^Each OR was adjusted for all other characteristics in the table

Data in bold shows significant differences

## Discussion

This study assessed the relationship between attribution of a causal role of smoking in three smoking-related diseases and study year, smoking status, age, and ethnicity among Estonian physicians from 1982 to 2014. Agreement with the statements that smoking is a cause of IHD, lung cancer, and chronic bronchitis was associated with study year, physicians’ smoking status and ethnicity.

While the nationwide coverage of physicians and the study period over three decades were major advantages of this survey, several potential limitations need addressing. First, the response rates to the surveys declined over time as has been reported in postal surveys among physicians worldwide [18]. Physicians are considered a difficult population from whom to collect survey data [19]. Low response rates can result in bias as non-respondents may be systematically different from respondents. However, the respondents were representative of the overall physician population in Estonia in terms of the male-female ratio, with a slightly higher proportion of respondents among female physicians. Second, these surveys relied on self-reports and this can result in bias as participants are less likely to be honest about measures relating to risk behaviours. Third, as the first study was conducted in the Soviet era, and some minor changes were made in study design and questions between studies, the findings of trends in this survey need to be interpreted with caution. Fourth, the size of some sub-groups (e.g ethnic group of non-Estonians by attitudes towards causal role of smoking in smoking-related diseases) per study year was small, and hence, the study estimates are not as precise as one might wish. Despite these shortcomings, several inferences can be drawn.

Compared with 1982, the proportion of respondents agreeing with the statements that smoking is a cause of IHD, lung cancer and chronic bronchitis was significantly higher among male and female physicians in 2002 and 2014. Already in 2002, the proportion of physicians who were unaware of the causal role of tobacco in lung cancer and chronic bronchitis was less than 2% among male and less than 1% among female physicians, and about 2% of male and female physicians did not consider tobacco to be a risk factor for IHD. In 2014, the percentage of non-awareness was less than 1.5% for all disease categories in both men and women. This could be attributed to the educational attainment of physicians, improvements in their knowledge about the health effects of smoking and a decline in their own smoking behaviours over three decades.

In 1982–2014, the age-standardized prevalence of current smoking decreased 2.8 times among male and 2.4 times among female physicians in Estonia and is lower than the prevalence of smoking among the general population and among the general population with higher education reflecting a ‘mature’ smoking epidemic in the country [20–23]. A similar decline in smoking among physicians has been reported in several developed countries [7, 20–25]. The prevalence of current smoking among 25–64-year-olds in the general population in Estonia was 56.7% for men and 25.8% for women in 2002 [26], and 40.5 and 22.2%, respectively, in 2014 [27]. The prevalence of current smoking among 25–64-year-olds with higher education in Estonia was 33.9% among men and 19.6% among women in 2002 [26], and 22.9 and 16.3%, respectively, in 2014 [27]. Thus, medical education and profession appears to have an impact on smoking as physicians know more about the harmful effects of smoking and they act as role models by their own behaviour in society as well as among their patients [4–6]. Unfortunately, no data are available for smoking rates among the general population in 1982 in Estonia. The decline in smoking among physicians as well as in the general population in Estonia might have been influenced by tobacco legislation as Estonia ratified the World Health Organization Framework Convention on Tobacco Control in 2005 and the same year the Estonian Parliament adopted a Tobacco Act to comply with the requirements laid down in the convention [28].

Compared with the female physicians who were current smokers, past smokers and those who had never smoked agreed significantly more with the attribution of smoking in all three smoking-related diseases. Compared with male physicians who were current smokers, past and never smokers agreed only with the statement that smoking is a cause of lung cancer. Attribution of a causal role of smoking in smoking-related diseases might be associated with the declining social acceptance of smoking in society, especially with legal restrictions on smoking in public spaces and mass media campaigns [28]. Also, earlier studies confirmed that compared to non-smokers, smokers systematically underestimate the health consequences of smoking [7–11, 29]. The stronger association of own smoking status with attribution of smoking as a cause in smoking-related diseases among females could be explained by gender role as a set of societal norms for females in society.

Compared to non-Estonians, agreement with the statements that smoking is a cause of IHD and chronic bronchitis was higher among Estonian male and female physicians, but attribution of a role of smoking in lung cancer was higher only among Estonian female doctors. Different attitudes towards the causal role of smoking in smoking-related diseases among Estonians and non-Estonians might be related to different social environments and differences in medical education as many non-Estonian physicians came to Estonia in adulthood and received their medical education outside Estonia, mainly in Russia. Nevertheless, one should not overestimate the findings concerning ethnic differences in attitudes towards the role of smoking in smoking-related diseases because of the small number of non-Estonian physicians who did not agree with this.

## Conclusion

Reported beliefs of physicians in Estonia were consistent with medical evidence and indicated little doubt about the smoking-related aetiology of IHD, lung cancer and chronic bronchitis. From 1982 to 2014, physicians’ attitudes towards the health risks of smoking improved in Estonia. However, their views on a causal role of smoking in smoking-related diseases was related to their own smoking habits and ethnicity. Past and never smokers were more likely to admit a causal role of smoking in lung cancer than those who did smoke. Agreement with statements about a causal role of smoking in IHD and chronic bronchitis was significantly higher among never and past smoking female physicians only. Compared to non-Estonians, agreement with these three statements was significantly higher among Estonians.

Although one-seventh of male physicians and one twentieth of female physicians in Estonia were current smokers in 2014 and the majority of physicians agreed on the causal role of smoking in smoking-related diseases, it is important that physicians’ smoking in Estonia continues its decline so that physicians can lead the way as tobacco control exemplars. A further decline in smoking among Estonian physicians would require special efforts targeted to physicians. Societal pressure from the national policy could further support a decline in the social acceptance of smoking in Estonia and continuing developments in medical education could provide continuing evidence-based knowledge concerning the health effects of smoking to Estonian physicians.

## References

[1] World Health Organization. WHO global report: mortality attributable to tobacco. World health Organization. 2012. http://www.who.int/tobacco/publications/surveillance/rep_mortality_attributable/en/. Accessed 16 Mar 2017.

[2] Centers for Disease Control and Prevention. Health effects of cigarette smoking. https://www.cdc.gov/tobacco/data_statistics/fact_sheets/health_effects/effects_cig_smoking/. Accessed 16 Mar 2017.

[3] Statistics Estonia. http://pub.stat.ee/px-web.2001/I_Databas/Population/03Vital_events/06Deaths/06Deaths.asp. Accessed 21 Mar 2017.

[4] Abdullah SA, Stillman FA, Yang L, Luo H, Zhang Z, Samet JM (2014). Tobacco use and smoking cessation practices among physicians in developing countries: a literature review (1987–2010). Int J Environ Res Public Health.

[5] Pipe A, Sorensen M, Reid R (2009). Physician smoking status, attitudes toward smoking, and cessation advice to patients: an international survey. Patient Educ Couns.

[6] World Health Organization. The role of health professionals in tobacco control. World health Organization. 2005. http://www.who.int/tobacco/resources/publications/wntd/2005/bookletfinal_20april.pdf. Accessed 17 Mar 2017.

[7] Pärna K, Rahu K, Barengo NC, Rahu M, Sandström PH, Jormanainen VJ (2005). Comparison of knowledge, attitudes and behaviour regarding smoking among Estonian and Finnish physicians. Soz Praventivmed.

[8] Lohur L, Pärna K (2016). Arstide suitsetamine, sellealased hinnangud ja tähelepanu pööramine patsientide suitsetamisele. [smoking habits, smoking related opinions and attitudes towards patients' smoking habits among physicians in Estonia]. Eesti Arst.

[9] Meshefedjian GA, Gervais A, Tremblay M, Villeneuve D, O'Loughlin J (2010). Physician smoking status may influence cessation counseling practices. Can J Public Health.

[10] Pärna K, Rahu K, Rahu M (2005). Smoking habits and attitudes towards smoking among Estonian physicians. Public Health.

[11] Waldron I, Lye D (1990). Relationships of teenage smoking to educational aspirations and parents' education. J Subst Abus.

[12] Davis RM (1993). When doctors smoke. Tob Control.

[13] Rahu M, Raudsepp J (1986). Teine Eesti NSV arstkonna suitsetamislevimuse ankeetküsitlus 1982. Aastal. [second questionnaire survey of smoking prevalence among physicians in Estonian republic of soviet union]. Nõukogude Eesti Tervishoid.

[14] Innos K, Rahu K, Baburin A, Rahu M (2002). Cancer incidence and cause-specific mortality in male and female physicians: a cohort study in Estonia. Scand J Public Health.

[15] Paapsi K, Pärna K (2015). Uuringu "Epidemioloogiline ja geneetiline tõendus tervshoiutöötajate suitsetamiskäitumise ja nikotiinisõltuvuse kohta" andmed kogutud. [data collection of the survey 'Epidemiological and genetic evidence of smoking behaviour and nicotine dependence among Estonian physicians' is finished]. Eesti Arst.

[16] Ahmad OP, Boschi-Pinto C, Lopez AD, Murray CJL, Lozano R, Inoue M. Age standardization of rates: a new WHO standard. GPE Discussion Paper Series: No. 31. Geneva: World Health Organization; 2001.

[17] Stata 12. StataCorp (2011). Stata statistical software: release 12.

[18] Cook JV, Dickinson HO, Eccles MP (2009). Response rates in postal surveys of healthcare professionals between 1996 and 2005: an observational study. BMC Health Serv Res.

[19] Cummings S, Savitz L, Konrad T (2001). Reported response rates to mailed physician questionnaires. Health Serv Res.

[20] Smith DR, Leggat PA (2007). An international review of tobacco smoking in the medical profession: 1974–2004. BMC Public Health.

[21] Smith DR, Leggat PA (2008). The historical decline of tobacco smoking among Australian physicians: 1964–1997. Tob Induc Dis.

[22] Smith DR, Wada K (2013). Declining rates of tobacco use in the Japanese medical profession, 1965–2009. J Epidemiol.

[23] Smith DR (2008). The historical decline of tobacco smoking among United States physicians: 1949–1984. Tob Induc Dis.

[24] Doll R, Peto R, Wheatley K, Gray R, Sutherland I (1994). Mortality in relation to smoking: 40 years' observations on male British doctors. BMJ.

[25] Barengo NC, Sandström PH, Jormanainen VJ, Myllykangas MT (2004). Changes in smoking prevalence among Finnish physicians 1990–2001. Eur J Pub Health.

[26] Kasmel A, Lipand A, Markina A (2003). Eesti täiskasvanud eanikkonna tervisekäitumise uuring, kevad 2002. health behaviour among Estonian adult population, spring 2002.

[27] Tekkel M, Veideman T. Eesti täiskasvanud rahvastiku tervisekäitumise uuring, 2014. In: Health behaviour among Estonian adult population, 2014. Tallinn: Tervise Arengu Instituut. p. 2015.

[28] Ministry of Social Affairs (2014). Green paper on tobacco policy.

[29] Willaing I, Jorgensen T, Iversen L (2003). How does individual smoking behaviour among hospital staff influence their knowledge of the health consequences of smoking?. Scand J Public Health.

